# Effect of chemical post-processing on the compressive strength of MSLA 3D printed orthodontic models: an in vitro comparative study

**DOI:** 10.2340/biid.v12.44904

**Published:** 2025-10-29

**Authors:** Neha Choudhary, Deepankar Bhatnagar, Swapna Sreenivasagan, Komal Yadav

**Affiliations:** a Department of Orthodontics Dentofacial Orthopaedics, MM College of Dental Sciences & Research, Maharshi Markandeshwar (Deemed to be University) Mullana, Ambala, Haryana, India; b Department of Orthodontics and Dentofacial Orthopaedics, Saveetha Dental College, Chennai, India

**Keywords:** MSLA 3D printing, compressive strength, post-processing, propylene glycol, orthodontic models

## Abstract

**Introduction:**

Three-dimensional (3D) printing, particularly Masked Stereolithography (MSLA), has emerged as a transformative technology in orthodontics for the fabrication of precise dental models. However, the mechanical integrity of these models, especially compressive strength, is critical during procedures such as thermoforming of clear aligners. This study aimed to evaluate the effect of different chemical post-processing methods on the compressive strength of MSLA-printed dental models.

**Materials and method:**

A total of 40 cylindrical resin samples (10 mm in height and 5 mm in diameter) were fabricated using an MSLA printer and divided into four groups (*n* = 10). Group 1: untreated group (acted as the control group), while Groups 2, 3, and 4 were treated with acetone, propylene glycol, and isopropyl alcohol, respectively. All treated samples were immersed in their respective chemicals for 5 min at room temperature. The compressive strength of each sample was measured utilising a Universal Testing Machine (UTM), and results were statistically analysed using Analysis of Variance (ANOVA) followed by Tukey’s *post hoc* test.

**Results:**

The compressive strength varied with statistical significance among the groups (*p* = 0.001). Compared to the untreated control group (107 ± 35 MPa), post-processing in propylene glycol (139 ± 48 MPa) or isopropyl alcohol (106 ± 10 MPa) resulted in statistically similar compressive strength, whereas post-processing in acetone (86 ± 19 MPa) led to significantly lower compressive strength. Furthermore, post-processing in propylene glycol resulted in significantly higher compressive strength than did post-processing in isopropyl alcohol or acetone. Propylene glycol showed greater variability, which makes its beneficial properties questionable.

**Conclusion:**

In conclusion, chemical post-processing significantly influences the compressive strength of MSLA-printed models. Acetone had a deleterious impact on compressive strength. Isopropyl alcohol proved to be an acceptable solvent. Propylene glycol showed large variability in results, warranting further investigation.

## Introduction

The advent of three-dimensional (3D) printing technology has revolutionised digital dentistry by facilitating rapid prototyping and fabrication of highly accurate dental models [1]. Among various 3D printing techniques, Masked Stereolithography (MSLA) has become increasingly favoured for its accuracy, precision, cost-effectiveness, and capacity to intricate fine surface details. Masked Stereolithography employs a light source that is filtered through a liquid-crystal display (LCD) screen to precisely cure photosensitive resin layers, resulting in the formation of complex dental structures with exceptional dimensional precision [2, 3].

In the workflow of MSLA 3D printing, post-processing plays a vital role that significantly impacts the mechanical and functional performance of the printed object. Generally, this process involves washing or cleaning the printed model with a solvent such as isopropyl alcohol to eliminate uncured resin, and then subjecting it to post-curing under ultraviolet light to enhance polymerisation [4, 5]. Nonetheless, variations in post-processing – especially regarding the selection and approach of chemical treatment – have a considerable impact on the mechanical properties of the final product, including compressive strength, which is crucial for the practical use of dental models, especially in areas such as aligner fabrication, prosthodontics, and occlusal assessment [6].

Previous studies have shown that failing to fully eliminate uncured resin or excessive exposure to solvents can negatively impact the strength of the material, surface roughness, and dimensional stability. Conversely, suitable chemical post-processing can strengthen polymer crosslinking and enhance the structural integrity of the printed model [7]. However, there is scarcity of literature comparing the impact of different chemical post-processing methods on the compressive strength of MSLA-printed dental models. The mechanical resilience of these models under compressive forces is essential for their application in clinical and laboratory environments, highlighting a significant area that justifies more investigations [8].

Therefore, this study aims to assess and compare the effect of different chemical post-processing liquids on the compressive strength of dental models fabricated using MSLA 3D printing technology. Understanding the relationship between post-processing techniques and mechanical performance will help optimise the workflow and ensure reliability in clinical outcomes.

## Materials and methods

**Figure 1:**
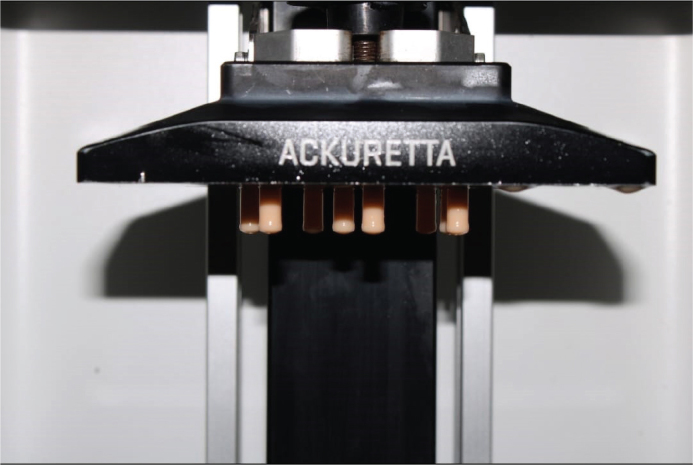
Masked stereolithography (MSLA) 3D printer (Ackuretta, Ackuretta Technologies Pvt. Ltd.)

**Figure 2:**
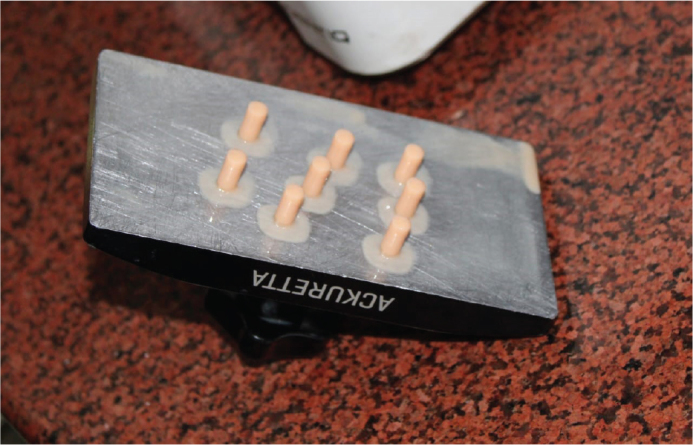
Cylindrical specimens fabricated for compressive strength testing.

This study involved the fabrication of 40 cylindrical test samples utilising a MSLA 3D printer (Ackuretta, Ackuretta Technologies Pvt. Ltd.). The photopolymer resin used for printing was Anycubic Standard LCD Molding Resin (Anycubic, China), designed for high-resolution applications in dental modelling. The digital models were developed using Computer-Aided Design (CAD) software and transformed into STL file format, which was subsequently processed using the manufacturer’s suggested slicing software to produce compatible print files for the MSLA printer (Figures 1 and 2).

**Figure 3:**
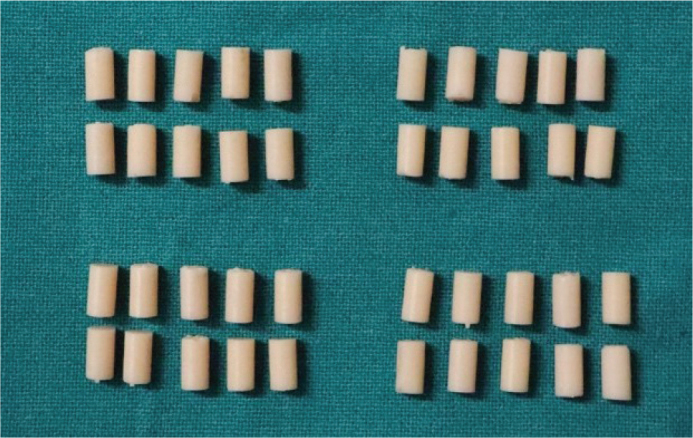
Cylinders of 5mm diameter and 10 mm length.

Each sample was designed in accordance with the EN ISO 604:2002 standard, which outlines the dimensions necessary for the determination of compressive properties of plastics. Cylindrical samples were fabricated with a diameter of 5 mm and a height of 10 mm as shown in Figure 3, achieving a diameter-to-height ratio (x/L) of 0.5, thereby fulfilling the standard requirement of a ratio ≥ 0.4 for valid compressive testing [9]. Following the printing process, the samples underwent a thorough cleaning and curing procedure in accordance with the manufacturer guidelines to ensure consistent polymerisation.

**Figure 4:**
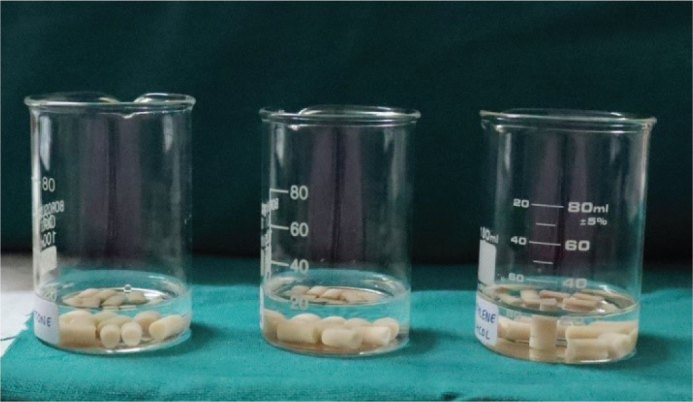
Group 2, 3, and 4 samples immersed in 99.97% acetone, 99.96% propylene glycol and 99% isopropyl alcohol, respectively for 5 min at room temperature.

The 40 samples were allocated randomly into four distinct groups, each group consisting of 10 samples. Group 1 acted as the control and did not undergo any chemical post-processing. Samples from Group 2 were immersed in 99.97% acetone (Tree Fit, ACS/Lab Grade) for a duration of 5 min at room temperature. Samples from Group 3 were immersed in 99.96% propylene glycol (Sharrets, USP/FCC/EP Grade), while Group 4 samples were immersed in 99% isopropyl alcohol (Cero, laboratory grade); both under identical conditions. All chemical baths were performed using 3.3 borosilicate glass beakers, chosen for their resistance to chemicals and ability to withstand thermal changes. After immersion, samples were taken out and allowed to air-dry in a pristine, dust-free environment for 10 min to remove any leftover solvent and ensure surface stabilisation (Figure 4).

Sample size was calculated using OpenEpi Version 3, incorporating a confidence level of 95% and a statistical power of 80%, based on effect sizes documented in previous studies of the compressive properties of MSLA-printed resins. The determined sample size provided adequate statistical power to identify difference between groups.

**Figure 5:**
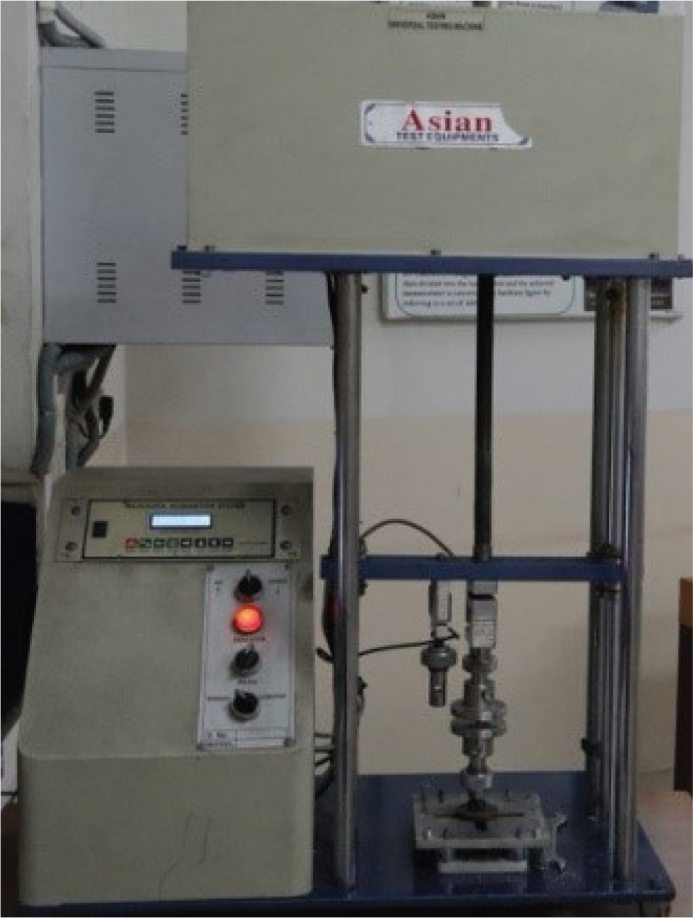
Universal Testing Machine setup for checking compressive strength.

Testing for compressive strength was conducted using a Universal Testing Machine (UTM) (Asian Test Equipments, ISO 9001:2008 Certified) (Figure 5). Each sample was orientated vertically along its longitudinal axis, and compression was exerted at a constant crosshead speed of 5 mm/min [10, 11]. The length of each sample was measured at 10.00 mm, accompanied by a cross-sectional area of 19.63 mm². The test was carried out in controlled laboratory conditions (25 ± 2°C), and persisted until the sample demonstrated structural failure. The maximum compressive load (measured in Newtons) was documented for each sample. The maximum compressive load of each sample was divided by the cross-sectional area to obtain the corresponding compressive strength (measured in MPa) for each sample. The outcomes were tabulated and analysed statistically for evaluating and comparing the effect of each chemical post-processing agent on the compressive strength of the MSLA-printed resin samples.

The analysis was conducted using IBM SPSS Statistics software (Version 21). For each group, descriptive statistics, including mean and standard deviation (SD), were calculated. The Shapiro-Wilk test was performed to confirm the normality of data distribution and Levene’s test was performed to assess the homogeneity of variances. One-way Analysis of Variance (ANOVA) test was conducted to identify statistically significant difference among the mean of compressive strength among the four groups. The ANOVA test indicated a significance result (*p* < 0.05), and subsequent *post hoc* pairwise comparisons were performed using Tukey’s (Honestly Significant Difference [HSD]) test to determine intergroup differences. A significance threshold of *p* < 0.05 was set for all the analyses.

## Result

**Figure 6:**
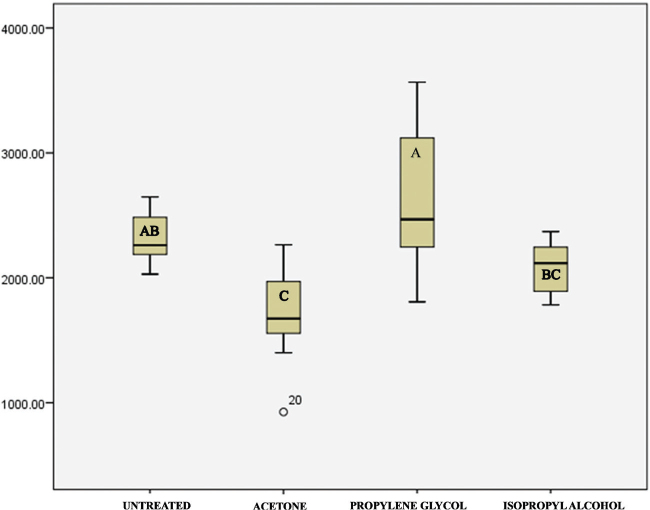
Histogram showing compressive strength (measured in Newtons) of the four groups. Letters denote the results of the Tukey’s post-hoc test.

The mean values of the maximum compressive load that the samples could withstand before undergoing structural failure are presented in Table 1 and Figure 6. The maximum compressive load varied between 1,689 and 2,626 N. The ANOVA found the compressive strength across the groups to vary with statistical significance (*F* = 10.340, *p* < 0.001), indicating that the type of solvent treatment significantly influences the mechanical performance of the printed resin material (Table 2).

**Table 1:** Descriptive analysis of the four groups showing maximum compressive load (measured in Newtons).

GROUP ACCORDING TO SOLVENT	*N*	Mean	Standard deviation	Standard error	95% confidence interval for mean	95% confidence interval for mean
					Lower bound	Upper bound
UNTREATED	10	2308.9770	198	62.89369	2166.7016	2451.2524
ACETONE	10	1689.3930	377	119.51714	1419.0264	1959.7596
PROPYLENE GLYCOL	10	2626.3460	611	193.21918	2189.2538	3063.4382
ISOPROPYL ALCOHOL	10	2084.3060	211	66.89811	1932.9720	2235.6400
Total	40	2177.2555	507	80.30882	2014.8156	2339.6954

**Table 2:** One way – ANOVA test for comparison of means among groups.

Source of variation	Sum of squares	df	Mean square	*F*	Sig.
Between Groups	4656822.592	3	1552274.197	10.340	.000
Within Groups	5404407.496	36	150122.430		
Total	10061230.088	39			

Table 3 shows the corresponding compressive strength values for each group. Propylene glycol group had the highest average compressive strength (138.85 MPa), but also the largest variability (SD = 48), while acetone treated group was found to have the lowest average compressive strength (86.03 MPa) (Tables 3 and 4).

**Table 3:** Descriptive analysis of the four groups showing compressive strength (measured in MPa).

GROUP ACCORDING TO SOLVENT	*N*	Mean	Standard deviation	Standard error	95% confidence interval for Mean	95% confidence interval for Mean
					Lower bound	Upper bound
UNTREATED	10	107.5950	35	11.13864	82.3976	132.7924
ACETONE	10	86.0390	19	6.08667	72.2700	99.8080
PROPYLENE GLYCOL	10	138.8520	48	15.28735	104.2696	173.4344
ISOPROPYL ALCOHOL	10	106.1520	10	3.40704	98.4447	113.8593
Total	40	109.6595	36	5.70905	98.1119	121.2071

**Table 4:** One way – ANOVA test for comparison of means among groups.

STRENGTH IN MPa					
Source of variation	Sum of squares	df	Mean square	*F*	Sig.
Between Groups	14266.948	3	4755.649	4.680	.007
Within Groups	36578.502	36	1016.070		
Total	50845.450	39			

Further elucidating the nature of these variations were *post hoc* intergroup comparisons applying Tukey’s HSD test (Table 5). Compared to the untreated control group, post-processing washing in propylene glycol and isopropyl alcohol resulted in statistically similar compressive strength, whereas post-processing in acetone led to a significant reduction in compressive strength. Furthermore, post-processing in propylene glycol resulted in significantly higher compressive strength than did post-processing in isopropyl alcohol or acetone.

**Table 5:** Results of the post-hoc (Tukey’s test) analysis.

(I) GROUP ACC. TO SOLVENT	(J) GROUP ACC. TO SOLVENT	Mean Difference (I-J)	Std. Error	Sig.
**UNTREATED**	**ACETONE**	**619.58400** ^*^	**173.27575**	**.005**
	PROPYLENE GLYCOL	–317.36900	173.27575	.276
	ISOPROPYL ALCOHOL	224.67100	173.27575	.571
**ACETONE**	**UNTREATED**	**–619.58400** ^*^	**173.27575**	**.005**
	**PROPYLENE GLYCOL**	**–936.95300** ^*^	**173.27575**	**.000**
	ISOPROPYL ALCOHOL	–394.91300	173.27575	.122
**PROPYLENE GLYCOL**	UNTREATED	317.36900	173.27575	.276
	**ACETONE**	**936.95300** ^*^	**173.27575**	**.000**
	ISOPROPYL ALCOHOL	542.04000^*^	173.27575	.017
ISOPROPYL ALCOHOL	UNTREATED	–224.67100	173.27575	.571
	ACETONE	394.91300	173.27575	.122
	PROPYLENE GLYCOL	–542.04000^*^	173.27575	.017

## Discussion

A paradigm shift in appliance fabrication, diagnostic planning, and treatment monitoring has been brought about by the incorporation of 3D printing technologies into orthodontic practice [12]. Because of its better resolution, smooth surface and affordability, MSLA printers have become a popular additive manufacturing method. Notwithstanding these benefits, issues with MSLA printed dental models’ mechanical performance-specifically, their compressive strength – continue to be a drawback in high-pressure applications such as clear aligner thermoforming [13, 14].

The purpose of our study was to examine how the compressive strength of resin-based models created using MSLA printing was affected by three distinct chemical post-processing agents: acetone, propylene glycol, and isopropyl alcohol; some commonly used post processing solvents [5, 15–18].

The result showed that the mechanical behaviour of the printed resin models was greatly impacted by the post-processing treatment. Calibration of compressive strength benchmarks for 3D‑printed dental models is still evolving. There is very little evidence available regarding compressive strength of printed models, which was a motivation to carry out this study. While no formal numeric standard exists, based on research, printed models should be able to withstand a minimum compressive force of 700 N. This would ensure that the model can withstand forces exerted by processes such as thermoforming [8, 13]. Our study found the compressive strength of all the groups to be above this threshold value, indicating that the models are adequate for most of the clinical procedures such as aligner fabrication. In addition, these models would not distort throughout the duration of orthodontic treatment and would serve well for record purposes as well.

This study found the compressive strength of propylene glycol treated group to be the highest. Other investigators have shown that propylene glycol has hygroscopic and somewhat viscous qualities, which may promote polymer cross-linking and interlayer adhesion during post-curing [19]. Propylene glycol may serve as a stabilising agent that improves microstructural cohesion and decreases surface imperfections [20]. However, the high SD found in the propylene glycol group indicates that treatment results may vary, perhaps as a result of uneven solvent absorption or interactions with resin chemistry. This suggests that in order to guarantee reproducibility, future standardisation of immersion time, concentration, and drying procedures is required. Hence, despite the somewhat higher mean compressive strength, it cannot be accurately concluded that propylene glycol is superior to other washing agents. Further investigation is needed into its impact on the compressive strength of models.

On the other hand, acetone is found to jeopardise the integrity of the polymer matrix. Acetone, a potent solvent with high volatility, can cause surface dissolution or encourage the development of microcracks at the interfacial layers, weakening the internal structure of the printed 3D models [21, 22]. These results are consistent with earlier studies showing that acetone degrades dental materials made of polymers [23].

The mechanical properties of the group treated with isopropyl alcohol were intermediate. Because it is effective at removing uncured resin, isopropyl alcohol is frequently used in dental 3D printing workflow for surface cleaning [24]. Although, its impact on internal mechanical properties hasn’t been thoroughly studied. The present study found that isopropyl alcohol treated samples had lower compressive strength than untreated models. This suggests that while isopropyl alcohol may be an effective outer surface cleaner, prolonged exposure may interfere with post-polymerisation or cause unreacted monomers to leach, resulting in reduced structural performance. Washing with isopropyl alcohol can negatively impact the mechanical quality and precision of the models if not handled properly and for the right duration of time. Short isopropyl alcohol washes (~5–10 min) are adequate to remove unreacted monomer and support good biocompatibility, without significantly compromising flexural strength or surface finish [8]. Longer washing time leads to significant deterioration of surface properties [25]. Alcohol diffuses into the resin, creating micro voids and weakening interchain bonds, leading to plasticisation and crazing [24]. For this study, a washing time of 5 min was selected so that all the objectives of post processing washing could be met without compromising the mechanical properties of the model.

The findings have important clinical ramifications, especially when it comes to the fabrication of clear aligners. The dental model must maintain its dimensional accuracy when subjected to localised pressure and heat during thermoforming. Insufficient compressive strength may cause the model to deform, which would compromise the final aligner’s geometric accuracy [13, 26]. As a result, improving post-processing procedures is crucial for maintaining the accuracy and efficacy of orthodontic appliances as well as for model longevity. Proper thermoforming requires that the printed model withstand the vacuum‐forming process. Studies have reported that 3D‑printed models, when post‑cured appropriately, withstood compressive loads of 495 N to 666 N in elastic deformation tests before fragmenting. Under all post‑curing conditions tested, the material sustained compressive loads in the range of 495–666 N. Thus, to avoid deformation during thermoforming, printed models should withstand compressive forces in this same range [27, 28].

In addition, the observed variation in compressive strength results highlights how crucial material solvent compatibility is in post-processing workflows. Propylene glycol’s variability points to inconsistent results, which call for more study on treatment standardisation, interaction mechanisms, and long term stability. Conversely, the negative effects of acetone obviously dissuade its use in post-processing procedures meant to improve mechanical qualities.

With all factors taken into account, this study offers insightful information about the choice of post-processing agents for dental models that are printed using MSLA. It warns against using common solvents such as acetone and isopropyl alcohol carelessly without proper mechanical validation. Exposure to acetone could result in surface dissolution or formation of microcracks within the printed matrix, which may significantly degrade the structural integrity. Isopropyl alcohol, while frequently used in 3D printing workflows, exhibited an intermediate mechanical impact. In addition, this study highlights that propylene glycol could have the potential to be a good washing agent. Propylene glycol has a variable effect on the compressive strength of the models. The large SD in the propylene glycol group suggests that it may have an unpredictable impact on compressive strength and more research is required to solidify its beneficial effects and establish propylene glycol as a suitable washing agent.

The information presented here lends credence to the theory that the functional performance of orthodontic models made using additive manufacturing is significantly influenced by chemical post-processing.

## Conclusion

This study demonstrated that chemical post-processing significantly influences the compressive strength of MSLA-printed dental models. Isopropyl alcohol emerged as a good washing solvent. Acetone treatment was found to be deleterious to compressive strength. Propylene glycol could make an acceptable solvent but large variability suggests that further investigation is needed to corroborate the benefits of this agent. The findings underscore the importance of solvent selection in post-processing protocols.
